# Baseline Depression-Like Behaviors in Wild-Type Adolescent Mice Are Strain and Age but Not Sex Dependent

**DOI:** 10.3389/fnbeh.2021.759574

**Published:** 2021-10-07

**Authors:** Ahmed Eltokhi, Barbara Kurpiers, Claudia Pitzer

**Affiliations:** ^1^Department of Pharmacology, University of Washington, Seattle, WA, United States; ^2^Interdisciplinary Neurobehavioral Core, Heidelberg University, Heidelberg, Germany

**Keywords:** depression, sex differences, tail suspension test, sucrose preference test, forced swim test, hyponeophagia test

## Abstract

Depression is a major neuropsychiatric disorder, decreasing the ability of hundreds of millions of individuals worldwide to function in social, academic, and employment settings. Beyond the alarming public health problem, depression leads to morbidity across the entire age including adolescence and adulthood. Modeling depression in rodents has been used to understand the pathophysiological mechanisms behind this disorder and create new therapeutics. Although women are two times more likely to be diagnosed with depression compared to men, behavioral experiments on rodent models of depression are mainly performed in males based on the assumption that the estrous cycles in females may affect the behavioral outcome and cause an increase in the intrinsic variability compared to males. Still, the inclusion of female rodents in the behavioral analysis is mandatory to establish the origin of sex bias in depression. Here, we investigated the baseline depression-like behaviors in male and female mice of three adolescent wild-type inbred strains, C57BL/6N, DBA/2, and FVB/N, that are typically used as background strains for mouse models of neuropsychiatric disorders. Our experiments, performed at two different developmental stages during adolescence (P22–P26 and P32–P36), revealed strain but no sex differences in a set of depression-related tests, including tail suspension, sucrose preference and forced swim tests. Additionally, the 10-day interval during this sensitive period uncovered a strong impact on the behavioral outcome of C57BL/6N and FVB/N mice, highlighting a significant effect of maturation on behavioral patterns. Since anxiety-related behavioral tests are often performed together with depression tests in mouse models of neuropsychiatric disorders, we extended our study and included hyponeophagia as an anxiety test. Consistent with a previous study revealing sex differences in other anxiety tests in adolescent mice, male and females mice behaved differently in the hyponeophagia test at P27. Our study gives insight into the behavioral experiments assessing depression and stresses the importance of considering strain, age and sex when evaluating neuropsychiatric-like traits in rodent models.

## Introduction

Depression is a long-lasting heterogeneous neuropsychiatric disorder and one of the most common mental diseases, which places a significant economic burden on public health and decreases individuals’ quality of life (Wittchen et al., 2011; Lim et al., 2012; Murray et al., 2013; Whiteford et al., 2013). It affects approximately 4.4% of the world’s population with an incidence rate above the rate of global population growth (Flux and Lowry, 2020). The symptoms include sadness, helplessness, guilt and loss of appetite, sexual desire, and interest in activities that once were pleasurable (for at least 2 weeks) along with recurrent thoughts of suicide (McCarter, 2008; American-Psychiatric-Association [APA], 2013). Because of the unique and complex features in addition to the subjective symptoms of human depression, the generation of valid and insightful depression models for the development of new therapeutic drugs is not straightforward. Still, different rodent models have shown high face validity to human depression and are used in preclinical studies. These depression-like behaviors can be induced in rodents by several means including genetic, environmental, chemical and pharmacological manipulation and brain lesions [for recent reviews, see (Flux and Lowry, 2020; Becker et al., 2021)]. Several tests are usually performed to assess distinct components of depression in rodent models. For example, the tail suspension and forced swim tests measure despair, the splash test measures apathy, while the sucrose preference test evaluates anhedonia (Eltokhi et al., 2018; Becker et al., 2021). Additionally, tests that measure anxiety including open field, elevated plus maze and light/dark compartment are often performed complementary to depression tests for the full characterization of rodent models of neuropsychiatric disorders (Belovicova et al., 2017).

The prevalence and clinical characteristics of depression differ between women and men with women suffering from depression nearly twice as frequently as men during lifetime, independently of race or ethnicity (Weissman and Klerman, 1977; Cyranowski et al., 2000; Andrade et al., 2003; Ford and Erlinger, 2004; Patten et al., 2006; Bromet et al., 2011; Salk et al., 2017). However, male rodent models have traditionally been used in genetic and pharmacological studies of depression. Performing behavioral experiments on male rodents is linked to the assumption of estrous-linked changes in the baseline behavioral activity in females. This concern may require testing female rodents at each of the four stages of the estrous cycle to generate reliable data, which may complicate the experimental design. On the other hand, neglecting the sex difference in depression-related experiments may provoke false interpretations of the results and prevents a successful translation of experimental data into the clinic. Thus, it is essential incorporating female rodents in behavioral, molecular and electrophysiological analyses to decipher the biology behind the sex bias of depression as a prerequisite for improving therapeutics. Unfortunately, so far, rodent models have yielded inconsistent results in different studies and often reported more depression-like symptoms in males than females (Kokras et al., 2009; Dalla et al., 2010; Barkus, 2013; Van den Hove et al., 2013; Najjar et al., 2018; Becker et al., 2021). By standardization of behavioral experiments and by taking into consideration factors that affect behavioral outcomes, the variability in results on sex differences can be likely reduced. Additionally, the sex differences of depression rodent models may also be induced by differences in the general performance and baseline activity of male and female wild-types in depression-related behavioral tests. To test these hypotheses, we investigated the baseline depression-like behaviors of both male and female wild-type mice in three standard behavioral tests, tail suspension, sucrose preference and forced swim tests. We performed our analysis in three inbred strains, C57BL/6N, DBA/2, and FVB/N, well-established to affect the behavioral outcome (McFadyen et al., 2003; Brooks et al., 2005; Moy et al., 2007; Peleh et al., 2019; Eltokhi et al., 2020, 2021), and typically used as background strains in mouse models of neuropsychiatric disorders. Furthermore, although behavioral studies are frequently performed in adult mice taking the advantage of easy handling and complex behaviors, we performed our analysis at two different developmental stages during adolescence since the onset of neuropsychiatric symptoms emerges mainly during adolescence, with more than 50% of adults with neuropsychiatric disorders receiving a diagnosis before 15 years of age (Kim-Cohen et al., 2003; Paus et al., 2008). Because behavioral results are known to be sensitive to small developmental progress during adolescence (Peleh et al., 2019; Eltokhi et al., 2020), we compared the results of these behavioral experiments between these two developmental stages in mice differing only by a few days in age.

Our work indicates that the performance of mice in depression-related behavioral tests during adolescence is mainly strain and age dependent with no obvious effect of sex. This outlines the drawbacks of using an individual strain in genetic and pharmacological studies of depression and highlights the benefits of using adolescent mice in characterizing rodent models of depression, which may reduce inconsistency of results between different laboratories.

## Materials and Methods

### Animals and Housing Conditions

Animals and housing conditions were similar to our previous studies (Eltokhi et al., 2020, 2021). The experiments were conducted in strict compliance with national and international guidelines for the Care and Use of Laboratory Animals. The behavioral analysis was carried out following the ARRIVE guidelines and was approved by the animal ethic committee of the (Regierungspräsidium Karlsruhe) Government of Baden Württemberg (G-101/16).

### Experimental Design and Groups

Depression and anxiety-related behavioral tests were carried out during the daylight cycle starting at 7 a.m. Mice were habituated to the behavioral room for half an hour before the start of the tests. We analyzed the depression-like behaviors in 2 cohorts of group-housed mice of both sexes with one cohort starting at P22 till P26 and the second starting at P32 till P46. The number of mice per cohort and the type of the behavioral experiments are listed (Table 1). The tail suspension test was the first test to be applied to each cohort. Starting the same day, we performed the sucrose preference test for 4 consecutive days. On day 5 the forced swim test was performed. For the anxiety-related hyponeophagia test, two other cohorts of mice were tested at P27 and P37.

**TABLE 1:** Mouse cohorts, number and age of the adolescent mice used in the behavioral test battery.

Cohorts	Strains	Mice (#)		Behavioral test at postnatal day (P#)
		Male ♂	Female ♀	
P22–26	C57BL/6N DBA/2 FVB/N	11 11 7	7 12 14	Tail suspension (P22) Sucrose preference (P22–25) Forced swim (P26)
P32–36	C57BL/6N DBA/2 FVB/N	10 9 7	14 9 11	Tail suspension (P32) Sucrose preference (P32–35) Forced swim (P36)
P27	C57BL/6N DBA/2 FVB/N	7 11 6	8 12 6	Hyponeophagia test
P37	C57BL/6N DBA/2 FVB/N	15 5 7	10 6 11	Hyponeophagia test

### The Behavioral Test Battery

#### The Tail Suspension Test

This test is useful in the screening of potential antidepressants and assessing depression-like behaviors in mice (Can et al., 2012). Each mouse was suspended to a rod by its tail with an adhesive tape at 55 cm above the surface. The latency for the first immobility and total immobility duration were measured during 6 min. An increased immobility duration or a reduced latency to first immobility are indicative of a depression-like phenotype. The test was videotaped and immobility time was analyzed by an independent observer.

#### The Forced Swim Test

This test was first introduced as a behavioral test to screen antidepressants (Porsolt et al., 1977; Porsolt, 1997). Mice were placed into a glass cylinder (20 cm in height, 14 cm in diameter) filled with water (24 ± 1°C) to a level that allowed mice to swim or float without their hind limbs or tails touching the bottom of the cylinder. The behavior of mice was monitored with the SYGNIS video tracker system (Sygnis Tracker 3) for 6 min and the immobility duration between 2 and 6 min was measured. Immobility was defined as a lack of swimming with only minimal movement of one hindlimb that was necessary to keep the head above water.

#### The Sucrose Preference Test

On day 1, the test was performed at P22 or P32 on single-housed mice in cages with two water bottles each. On the following day (day 2), both bottles were removed and changed with a bottle filled with water and a second one filled with a 1% sucrose solution. Both bottles were weighed before placing them into the cage. On day 3, bottles were weighed to determine the liquid consumption during the previous 24 h. Bottles were then refilled and weighed and placed into the cage with an alternated position of the sucrose vs. water bottle to avoid place preference. On day 4, bottles were weighed. The sucrose preference index was calculated as the average consumed sucrose across the last 2-day period divided by the average volume of total consumed liquid (average water plus average sucrose solution).

#### The Hyponeophagia Test

The hyponeophagia test measures the reduction in feeding in response to a novel environment and a portion of new food or drink, since they induce anxiety, resulting in a delayed food intake. Therefore, this test can be used for the assessment of emotionality and anxiety. To perform the test, we followed the protocol suggested by Deacon (2011) with minor modifications. 1 day prior to testing, food was rationed, and mice were given small pellets of 1 g per mouse. A food well filled with 1:1 milk diluted with water was placed under an inverted transparent jar (15 cm diameter). The mouse was carefully placed under the jar facing away from the food well. The latency to lick the milk continuously for >2–3 s during 2 min was measured. After finishing trial 1, the mouse was placed back into its home cage. After 3 min in the home cage, the mouse was placed again under the jar for another 2 min as trial 2, and the latency to lick the milk was measured.

### Statistical Analysis

Two-way ANOVA was used with sex and genotype as the two factors. This was followed by Tukey’s *post hoc* test for multiple comparisons to determine differences between the three strains C57BL/6N, DBA/2, and FVB/N and Bonferroni correction to check differences between males and females within each strain. To compare the two developmental stages (P22–26) and (P32–36) within each strain, two-way ANOVA was used with sex and age as the two factors. A *P* value ≤ 0.05 was considered statistically significant. To unravel the effect of the interaction between strain, sex, and age, three-way ANOVA was performed using a confidence interval of 95% and a tolerance of 0.0001. A Pr(>*F*) less than 0.05 was considered statistically significant. Statistical analysis was performed using GraphPad Prism 7 and Microsoft Office Excel including the XLSTAT software. The respective numbers of male and female mice per cohort are described in Table 1.

## Results

### Baseline Depression-Like Behaviors in P22–26 Mouse Cohort

In the first set of experiments, we tested the baseline depression-like behaviors in the wild-type mice using three tests that are well known to assess depression in rodent models. Both forced swim (Porsolt et al., 1977; Porsolt, 1997) and tail suspension tests (Steru et al., 1985) measure the immobility of rodents as an indication of despair when they cannot escape from an aversive situation. Additionally, we performed the sucrose preference test as a reward-based test to assess anhedonia or decreased ability to experience pleasure, as a core symptom of depression (Willner et al., 1987; Papp et al., 1991).

**FIGURE 1:**
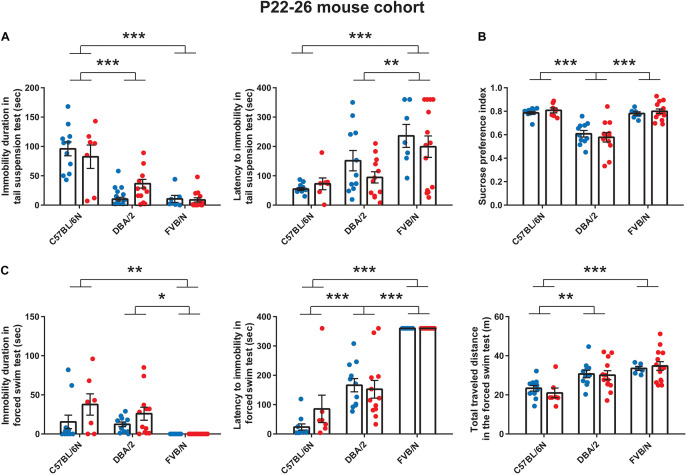
Baseline depression-like behaviors in P22–26 mouse cohort. **(A)** In the tail suspension test, C57BL/6N mice showed a significant increase in the baseline immobility duration compared to both DBA/2 and FVB/N mice. Additionally, FVB/N mice showed a significant increase in the latency to first immobility compared to both C57BL/6N and DBA/2 mice. **(B)** In the sucrose preference test, DBA/2 mice showed a lower sucrose preference index than both C57BL/6N and FVB/N mice. **(C)** In the forced swim test, FVB/N mice showed lower immobility duration and increased latency to first immobility than C57BL/6N and DBA/2 mice. C57BL/6N showed a decreased total traveled distance compared to DBA/2 and FVB/N mice. In **(A–C)**, no sex difference was revealed within any of the aforementioned strains. Blue and red dots represent males and females, respectively. Two-way ANOVA followed by Tukey *post hoc* test,*p ≤ 0.05, **p ≤ 0.01, and ***p ≤ 0.001. Error bars indicate the standard error of the mean (SEM).

We started our experiments by investigating the depression-like behaviors in male and female mice from a very young cohort (P22–P26). At P22, the tail suspension test revealed a significant increase in the baseline immobility duration of C57BL/6N mice compared to both DBA/2 and FVB/N mice (*P* < 0.0001 vs. DBA/2 and DBA/2; Figure 1A). Additionally, FVB/N mice showed increased latency to first immobility compared to both C57BL/6N and DBA/2 mice (*P* < 0.0001 vs. C57BL/6N, *P* = 0.007 vs. DBA/2; Figure 1A). For the sucrose preference test, DBA/2 mice showed an increased baseline anhedonia-like behaviors by having a significantly decreased sucrose preference index compared to both C57BL/6N and FVB/N mice (*P* < 0.0001 vs. C57BL/6N and FVB/N; Figure 1B). In the forced swim test at P26, FVB/N mice showed no immobility at all, and C57BL/6N mice showed a significant decreased latency to first immobility and total traveled distance compared to both DBA/2 and FVB/N mice (Immobility duration: C57BL/6N vs. DBA/2: *P* = 0.78, C57BL/6N vs. FVB/N: *P* = 0.004, DBA/2 vs. FVB/N: *P* = 0.015; Latency to first immobility: C57BL/6N vs. DBA/2: *P* < 0.0001, C57BL/6N vs. FVB/N: *P* < 0.0001, DBA/2 vs. FVB/N: *P* < 0.0001; Traveled distance: C57BL/6N vs. DBA/2: *P* = 0.001, C57BL/6N vs. FVB/N: *P* < 0.0001, DBA/2 vs. FVB/N: *P* = 0.16; Figure 1C). Interestingly, comparing the performance of male and female mice within each strain revealed no significant difference in their behaviors in any of the aforementioned tests (Table 2).

**TABLE 2:** List of the *P* values of the comparison between male and female mice of C57BL/6N, DBA/2, and FVB/N strains in the behavioral test battery.

	C57BL/6N	DBA/2	FVB/N	C57BL/6N	DBA/2	FVB/N
		
		P22–26 cohort			P32–36 cohort	
Tail suspension test: Immobility duration	0.979	0.087	>0.999	0.160	>0.999	>0.999
Tail suspension test: Latency to immobility	>0.999	0.470	>0.999	>0.999	>0.999	0.321
Sucrose preference index	>0.999	>0.999	>0.999	>0.999	>0.999	>0.999
Forced swim test: Immobility duration	0.117	0.422	>0.999	0.401	>0.999	>0.999
Forced swim test: Latency to immobility	0.243	>0.999	>0.999	>0.999	>0.999	>0.999
Forced swim test: Total traveled distance	>0.999	>0.999	>0.999	0.581	0.996	>0.999
	**P27 cohort**			**P37 cohort**		
Hyponeophagia test trial 1: Latency to drink	*0.006*	*0.041*	>0.999	>0.999	>0.999	>0.999
Hyponeophagia test trial 2: Latency to drink	*0.014*	*0.027*	0.550	>0.999	0.721	>0.999

*The italic indicates significant results.*

### Baseline Depression-Like Behaviors in P32–36 Mouse Cohort

**FIGURE 2:**
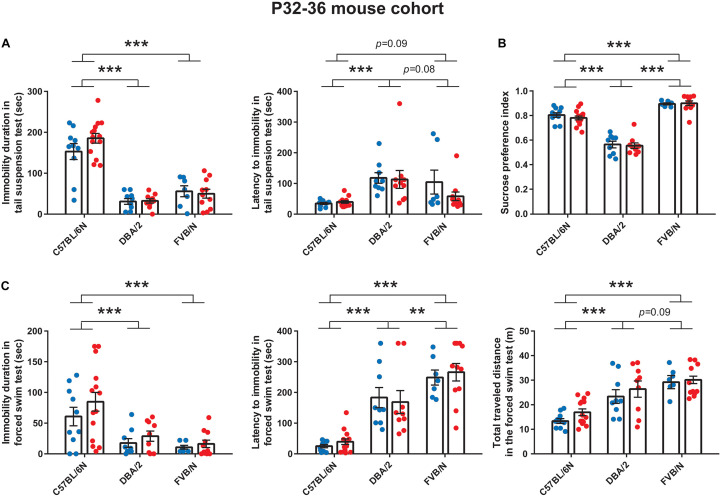
Baseline depression-like behaviors in P32–36 mouse cohort. **(A)** In the tail suspension test, C57BL/6N mice showed a significant increase in the baseline immobility duration compared to both DBA/2 and FVB/N mice. C57BL/6N mice showed a significantly decreased latency to first immobility compared to DBA/2 and borderline decreased latency compared to FVB/N mice. **(B)** In the sucrose preference test, DBA/2 mice showed an increased baseline anhedonia-like behaviors by having a significantly decreased sucrose preference index compared to both C57BL/6N and FVB/N mice. Moreover, FVB/N mice showed an increased sucrose preference index compared to C57BL/6N mice. **(C)** In the forced swim test, C57BL/6N mice showed an increased immobility duration, a decreased latency to first immobility and a decreased total traveled distance compared to both DBA/2 and FVB/N mice. In **(A–C)**, no sex difference was revealed within any of the aforementioned strains. Blue and red dots represent males and females, respectively. Two-way ANOVA followed by Tukey *post hoc* test, **p ≤ 0.01, ***p ≤ 0.001. Error bars indicate the standard error of the mean (SEM).

Similar to the results at P22 (Figure 1A), the tail suspension test at P32 revealed a significant increase in the baseline immobility duration of C57BL/6N mice compared to both DBA/2 and FVB/N mice (*P* < 0.0001 vs. DBA/2 and FVB/N; Figure 2A). Additionally, C57BL/6N mice showed a significantly decreased latency to first immobility compared to DBA/2 and borderline decreased latency compared to FVB/N mice (*P* = 0.0001 vs. DBA/2, *P* = 0.09 vs. FVB/N; Figure 2A). For the sucrose preference test, DBA/2 mice showed an increased baseline anhedonia-like behaviors by having a significantly decreased sucrose preference index compared to both C57BL/6N and FVB/N mice (*P* < 0.0001 vs. C57BL/6N and FVB/N; Figure 2B). Moreover, FVB/N mice showed an increased sucrose preference index compared to C57BL/6N mice (*P* < 0.0001). In the forced swim test at P36, C57BL/6N mice showed an increased immobility duration, a decreased latency to first immobility and a decreased total traveled distance compared to both DBA/2 and FVB/N mice (Immobility duration: C57BL/6N vs. DBA/2: *P* = 0.001, C57BL/6N vs. FVB/N: *P* < 0.0001, DBA/2 vs. FVB/N: *P* = 0.73; Latency to first immobility: C57BL/6N vs. DBA/2: *P* < 0.0001, C57BL/6N vs. FVB/N: *P* < 0.0001, DBA/2 vs. FVB/N: *P* = 0.004; Traveled distance: C57BL/6N vs. DBA/2: *P* < 0.0001, C57BL/6N vs. FVB/N: *P* < 0.0001, DBA/2 vs. FVB/N: *P* = 0.09; Figure 2C). Similar to the P22–26 mouse cohort, the P32–36 mouse cohort did not reveal a significant sex difference in the depression-like behaviors in any of the aforementioned tests (Table 2).

### Comparison Between the P22–26 and P32–36 Mouse Cohorts Within C57BL/6N, DBA/2, and FVB/N Strains

**FIGURE 3:**
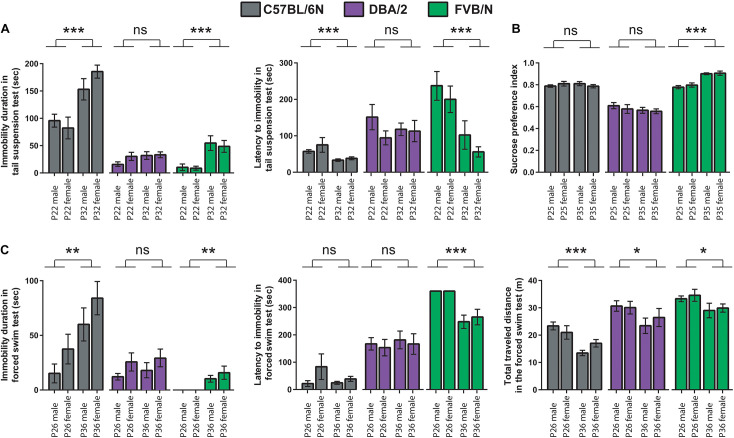
Comparison between the P22–26 and P32–36 mouse cohorts within C57BL/6N, DBA/2, and FVB/N strains. **(A)** In the tail suspension test, P32 C57BL/6N and P32 FVB/N mice showed a significantly increased immobility duration and decreased latency to first immobility compared to P22 C57BL/6N and P22 FVB/N mice, respectively. **(B)** In the sucrose preference test, FVB/N mice showed an increased sucrose preference index in older compared to younger mice with no effect of age on C57BL/6N and DBA/2 strains. **(C)** In the forced swim test, P36 C57BL/6N and P36 FVB/N mice showed a significantly increased immobility duration compared to P26 C57BL/6N and P26 FVB/N mice, respectively. For the latency to the first immobility, only P36 FVB/N mice showed a significantly decreased latency to the first immobility compared to P26 mice. For the total traveled distance, all three strains showed a decreased traveled distance in older compared to a younger age. Two-way ANOVA followed by Tukey *post hoc* test,*p ≤ 0.05, **p ≤ 0.01, and ***p ≤ 0.001. Error bars indicate the standard error of the mean (SEM).

To test whether the age difference of 10 days can affect the baseline depression-like behaviors during adolescence, results of the behavioral test battery were compared between the two cohorts. In the tail suspension test, P32 C57BL/6N and P32 FVB/N mice showed a significantly increased immobility duration compared to P22 C57BL/6N and P22 FVB/N mice, respectively (C57BL/6N: *P* < 0.0001, FVB/N: *P* < 0.0001; Figure 3A). In contrast, P32 C57BL/6N and P32 FVB/N mice showed a significantly decreased latency to first immobility compared to P22 C57BL/6N and P22 FVB/N mice, respectively (C57BL/6N: *P* = 0.0005, FVB/N: *P* = 0.0004). On the other hand, no difference in the immobility duration (*P* = 0.17) or latency to first immobility (*P* = 0.78) was found between P32 and P22 DBA/2 mice (Figure 3A). This discrepancy in the effect of age on strain is evident by the significant Pr(>*F*) value in the three-way ANOVA test [Strain^∗^Age: Pr(>*F*) < 0.0001 for both the immobility duration and latency to first immobility] (Table 3).

**TABLE 3:** List of the Pr(>*F*) after performing three-way ANOVA test to assess the effect of the interaction between strain, sex and age on the depression-like behavior.

**Tail suspension test: Immobility duration**		**Forced swim test: Immobility duration**	
Strain*Age	*<0.0001*	Strain*Age	*0.010*
Strain*Sex	0.447	Strain*Sex	0.372
Age*Sex	0.447	Age*Sex	0.901
**Tail suspension test: Latency to immobility**		**Forced swim test: Latency to immobility**	
Strain*Age	*<0.0001*	Strain*Age	*0.004*
Strain*Sex	0.295	Strain*Sex	0.313
Age*Sex	0.699	Age*Sex	0.672
**Sucrose preference index**		**Forced swim test: Total traveled distance**	
Strain*Age	*<0.0001*	Strain*Age	0.785
Strain*Sex	0.616	Strain*Sex	0.991
Age*Sex	0.655	Age*Sex	0.195

*The italic indicates significant results.*

For the sucrose preference test, FVB/N mice showed an increased sucrose preference index in older compared to younger mice with no effect of age on C57BL/6N and DBA/2 strains (C57BL/6N: *P* = 0.96, DBA/2: *P* = 0.32, FVB/N: *P* < 0.0001; Figure 3B), suggesting a sensitivity of the strain effect to mouse age [Strain^∗^Age: Pr(>*F*) < 0.0001] (Table 3). In the forced swim test, P36 C57BL/6N and P36 FVB/N mice showed a significantly increased immobility duration compared to P26 C57BL/6N and P26 FVB/N mice, respectively (C57BL/6N: *P* = 0.008, DBA/2: *P* = 0.52, FVB/N: *P* = 0.002; Figure 3C). For the latency of the first immobility, only P36 FVB/N mice showed a significantly decreased latency to the first immobility compared to P26 mice (*P* < 0.0001; Figure 3C). These results suggest different effects of age on the behavioral outcome in the forced swim test depending on which strain is used [Immobility duration: Strain^∗^Age: Pr(>*F*) = 0.01], latency of the first immobility: Strain^∗^Age: Pr(>*F*) = 0.004 (Table 3). In contrast, all three strains showed a similar decrease in the traveled distance in older compared to younger age (C57BL/6N: *P* < 0.0001, DBA/2: *P* = 0.04, FVB/N: *P* = 0.04; Figure 3C), and suggesting no interaction between strain and age [Strain^∗^Age: Pr(>*F*) = 0.785] (Table 3).

### Anxiety-Related Hyponeophagia Test at P27 and P37

**FIGURE 4:**
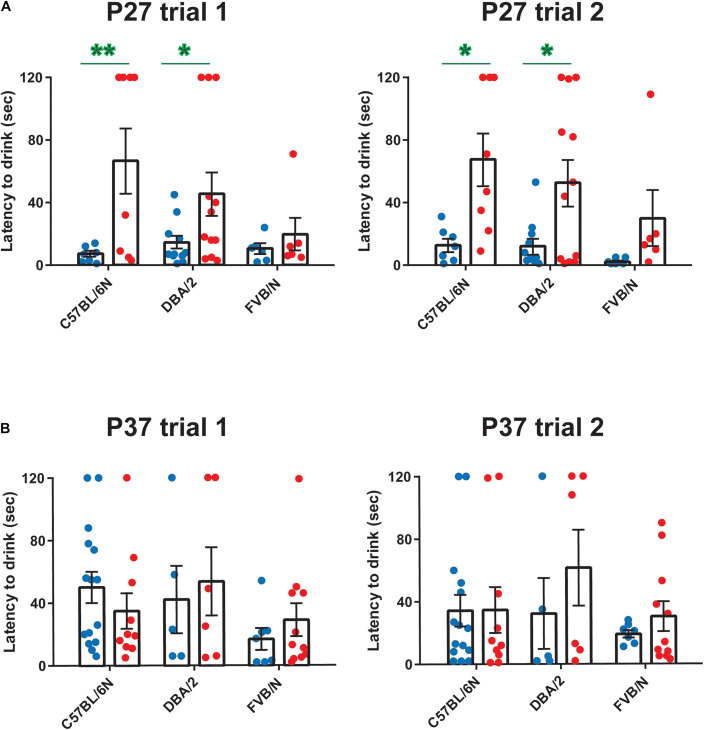
Anxiety-related hyponeophagia test at P27 and P37. Female C57BL/6N and DBA/2 mice showed an increased latency to drink the diluted milk compared to male mice in both trials 1 and 2 at P27 **(A)** but not at P37 **(B)**. At both P27 and P37, no strain difference was found in the latency to drink the diluted milk. The green asterisk indicates a significant difference between males and females. Two-way ANOVA followed by Tukey *post hoc* test,*p ≤ 0.05, **p ≤ 0.01. Blue and red dots represent males and females, respectively. Error bars indicate the standard error of the mean (SEM).

Since anxiety-related behavioral tests are often performed together with depression tests in mouse models of neuropsychiatric disorders (Cryan and Mombereau, 2004; Millstein and Holmes, 2007; Krishnan and Nestler, 2011; Jung et al., 2014; Belovicova et al., 2017; Sokolowska et al., 2021), we extended our study and investigated sex differences in an anxiety test, the hyponeophagia test. Consistent with our previous study revealing sex differences in other anxiety tests in adolescent mice (Eltokhi et al., 2020), female C57BL/6N and DBA/2 mice showed an increased latency to drink the diluted milk compared to male mice at P27 (Trial 1: C57BL/6N: *P* = 0.006, DBA/2: *P* = 0.041 FVB/N**:** *P* = 0.99; Trial 2: C57BL/6N: *P* = 0.014, DBA/2: *P* = 0.027, FVB/N**:** *P* = 0.55; Figure 4A and Table 2). In contrast, this sex effect was not present at P37, highlighting the effect of a 10-day interval effect on the behavioral outcome (Figure 4B and Table 2). At both P27 and P37, no strain difference was found in the latency to drink the diluted milk (P27 trial 1: C57BL/6N vs. DBA/2: *P* = 0.75, C57BL/6N vs. FVB/N: *P* = 0.20, DBA/2 vs. FVB/N: *P* = 0.44; P27 trial 2: C57BL/6N vs. DBA/2: *P* = 0.75, C57BL/6N vs. FVB/N: *P* = 0.16, DBA/2 vs. FVB/N: *P* = 0.37; P37 trial 1: C57BL/6N vs. DBA/2: *P* = 0.95, C57BL/6N vs. FVB/N: *P* = 0.23, DBA/2 vs. FVB/N: *P* = 0.24; P37 trial 2: C57BL/6N vs. DBA/2: *P* = 0.62, C57BL/6N vs. FVB/N: *P* = 0.78, DBA/2 vs. FVB/N: *P* = 0.33).

## Discussion

The global increased prevalence of depression in women compared to men suggests that the differential risk is highly dependent on biological sex differences rather than race, culture or other potentially confounding social and economic factors (Albert, 2015). As the onset of depression in women peaks in their reproductive years, the increased prevalence of depression may be explained in part by sex hormones. Indeed, the female hormonal fluctuation during puberty, menstruation, pregnancy, and menopause is a trigger for depression (Albert, 2015). The risk of depression increases during the perimenopausal transition (Cohen et al., 2006), with hormone replacement therapy being effective in the prevention of postmenopausal depression in women (Gordon and Girdler, 2014). Androgens seem to have anxiolytic properties whereas estrogens receptors (ER) activation has opposite consequences with ERα having largely anxiogenic-like properties and ERβ serving to generate anxiolytic-like effects (Borrow and Handa, 2017).

Here we evaluated depression-like behaviors in adolescent mice at two different developmental stages. Our previous studies unraveled that sex differences during adolescence are apparent in certain strains and behaviors such as general activity, anxiety and cognitive function (Eltokhi et al., 2020) as also shown for adult mice (Tarantino et al., 2000). Given the divergent susceptibility of males and females to depression, there is an urgent need for tackling the effect of sex in rodents on behavioral tests assessing depression-like behaviors. However, in all the tests commonly used to assess depression-like behaviors in rodents, we did not find any sex difference in C57BL/6N, DBA/2, or FVB/N strains at these two developmental stages during adolescence. Since we investigated the baseline depression-like behaviors only in young mice before puberty corresponding to P42 in mice (Taft et al., 2006; Dutta and Sengupta, 2016), with no severe effect of sex hormones, we interpret the finding that the effect of sex may become apparent only in adult mice. Indeed, a sex difference in the prevalence of patients with major depressive disorder appears mainly after puberty (Bebbington et al., 1998; Merikangas et al., 2010; Mehta et al., 2013). In adult C57BL/6J mice, the baseline immobility durations in the tail suspension tests were lower in males than females (Liu and Gershenfeld, 2001). Thus, the biological maturation occurring during puberty along with the intensification of sex-specific social roles may be a major key of sex differences in depression (Dalla et al., 2010). On the other hand, a more recent piece of evidence suggests that the sex difference in depression begins in childhood and becomes more pronounced during adolescence (Breslau et al., 2017). Irrespective of these analyses in humans, inconsistent results for depression and anxiety-like behaviors have been reported when comparing male and female adolescent rodents to adults (Slawecki, 2005; Doremus et al., 2006; Hefner and Holmes, 2007; Doremus-Fitzwater et al., 2009; Martínez-Mota et al., 2011). Thus, further developmental and longitudinal studies in rodents should be performed to assess the baseline depression in males and females along different developmental stages. Since the onset of puberty is strain dependent (Pintér et al., 2007), a rough dating of puberty of rodent models should be employed using various markers including the vaginal opening, first vaginal cornification, onset of cyclicity in females and balanopreputial separation in males.

Strain differences in behavioral tests assessing neuropsychiatric-like phenotypes in adolescent mice were repeatedly reported by our group (Peleh et al., 2019; Eltokhi et al., 2020, 2021). The contribution of genetic factors specifically in the depression-related behaviors of adult inbred rodents as well as in the efficacy of antidepressant drug treatment has been previously shown (Vaugeois et al., 1997; Liu and Gershenfeld, 2001; Lucki et al., 2001). In our study, the baseline depression-like behaviors differed between different strains. In both tail suspension and forced swim tests, C57BL/6N mice showed the highest immobility duration and the lowest latency to first immobility compared to DBA/2 and FVB/N. Interestingly, FVB/N mice at P26 did not show any immobility in the forced swim test. This behavior can be explained by their severe hyperactivity as previously reported (Eltokhi et al., 2020). These data are consistent with a former study showing adult FVB/NJ mice having the least baseline immobility duration in the forced swim test, DBA/2J being intermediate and C57BL/6J showing the highest immobility duration (Lucki et al., 2001). On the other hand, adolescent FVB/N mice showed the highest sucrose preference index, highlighting the suitability of this test to be performed on mice with FVB/N background. In contrast, DBA/2 mice showed low ability to perform the sucrose preference test and had a high baseline anhedonic level, which may mask the depression behavior in mouse models of neuropsychiatric disorders and should be taken into consideration in designing the experiments and analyzing the data. Nonetheless, DBA/2 can be a good strain to test the effect of anti-depressant drugs. Thus, the choice of the strain should be related to the specific scientific question being asked.

As our previous studies uncovered strong changes in behavioral outcomes including activity, anxiety and social interaction in a small developmental window during adolescence (Eltokhi et al., 2020, 2021), we here extended these findings assessing despair by tail suspension and forced swim tests. Both C57BL/6N and FVB/N mice showed an increase in immobility within 10 days of adolescence. For the sucrose preference test, the effect of the 10-day interval was only apparent in FVB/N mice. Interestingly, DBA/2 mice did not show any difference in the behavioral outcome at both time points, highlighting a direct correlation of the effect of age to specific strains. One limitation of our study is that the same mice were tested in a series of depression-related tests, which may have caused earlier tests to affect subsequent performance in later tests. To mitigate this possibility, we ordered the tests in such a way that the two stressful tests, tail suspension and forced swim tests, were not consecutive but separated by the sucrose preference test that was performed in the mouse homecage. Still, the single housing during the sucrose preference test may have also induced some levels of stress on mice, which may have affected their behaviors in the forced swim test. However, we believe that the possible effect of single housing was not strong as indicated by the similar pattern of behaviors in both tail suspension and forced swim tests at both developmental stages, with C57BL/6N mice showing increased immobility durations in both tests. Our findings suggest that taking the effect of age and strain into account will decrease variabilities and inconsistencies and increase the reproducibility of behavioral results between different laboratories.

Anxiety-related behavioral tests are often performed together with depression tests in mouse models of neuropsychiatric disorders since anxiety usually co-morbids with depression (Cryan and Mombereau, 2004; Millstein and Holmes, 2007; Krishnan and Nestler, 2011; Jung et al., 2014; Belovicova et al., 2017; Sokolowska et al., 2021). Several behavioral tests based on the conflict between competing behaviors of exploring novel environments and avoiding potential threatening situations have been validated to assess anxiety, including the elevated plus maze (Pellow et al., 1985; Lister, 1987), open field (Treit and Fundytus, 1988), and light/dark box (Crawley and Goodwin, 1980; Crawley, 1981; Blumstein and Crawley, 1983), where our previous studies revealed sex differences in the open field and elevated plus maze tests with female C57BL/6N and FVB/N mice being less anxious (Eltokhi et al., 2020). To unravel whether different anxiety paradigms tax distinct aspects of anxiety, we here investigated the sex difference in another anxiety-related test, the hyponeophagia test. Female C57BL/6N and DBA/2 but not FVB/N mice showed higher latency to consume the unfamiliar drink in an unfamiliar arena compared to males, reflecting increased bait shyness as an indication of anxiety. This discrepancy of results of males vs. females in different anxiety tests suggests that a battery of different tests should be used in studies of anxiety-related behaviors to draw the full picture of mouse phenotype (van Gaalen and Steckler, 2000).

Taken together, since depression is a complex disorder with several endophenotypes including despair, anhedonia and apathy, a complete test battery combining the well-established, robust behavioral tests should be employed when testing rodent models of neuropsychiatric disorders or investigating the effect of anti-depressant drugs. Sex, strain and age are suggested to have different effects on distinct behavioral tests. Therefore, performing a complete test battery will provide important information per animal/drug that cannot be covered in separate behavioral studies. Notably, there should be at least a 1-day rest period between each of the tests in the behavioral battery, with some rodent strains requiring even longer durations (Lad et al., 2009). Some paradigms currently used for assessing antidepressant and/or depression-like behaviors in mice are still questionable. For example, in recent years, there is a trend to interpret the transition from swimming to immobility in the forced swim test as a coping mechanism with inescapable stressors, rather than an indication of despair (Molendijk and de Kloet, 2019). However, the majority of researchers still qualify the rodent’s floating response as a depression-like behavior since the persistence of coping with inescapable stressors may indeed enhance the vulnerability to depression (Molendijk and de Kloet, 2019). These different scientific perspectives confirm the necessity of performing a complete test battery in order to draw a full picture of the depression-like behavior.

Finally, we want to touch on the issue of reproducibility of behavioral results in depression-related studies. One potential factor of discrepancy is the slightly different protocols between laboratories. Worth noticing, behavioral tests may cause stress and put unwanted burdens on rodents. To this end, the order of tests on a specific cohort can also play a role in the variabilities between the results of different laboratories. Moreover, different rodent strains and sex dependence of the stress response frequently result in apparent discrepancies in published data. However, our study, exemplified by trials 1 and 2 in latency to drink (Figure 4), demonstrated that consistency of results can be easily achieved by adequate test settings. Furthermore, during adolescence, no sex differences were found. This finding does not exclude an impact of sex in other developmental stages. However, in studies where sex differences are suggested to provoke an additional complication in depression analysis, adolescent mice may open a window for clearly identifying such effects since the general performance and baseline depression activity of male and female wild-type mice are similar.

Thus, strain, age and sex should be taken into account when analyzing neuropsychiatric disorders in mouse models. Without question, optimization and standardization of depression-related tests in rodent models will help in understanding pathophysiological mechanisms and in identifying novel targets for depression treatment.

## Conclusion

To our knowledge, this is the first study investigating sex, age and strain effects on the baseline depression-like behaviors. We confirmed that genetic strain differences and even small differences in developmental stage are important determinants of depression-related behavioral outcomes. We suggest using adolescent mice, at least in the three investigated depression-related behavioral tests, to reduce variability and inconsistency between different laboratories. Nonetheless, sex differences in mice still need a thorough evaluation throughout their lifetime. Taken together, our behavioral studies in adolescent mice can be used as a guiding platform for the choice of the most suitable combinations of assays and appropriate strain, age and sex selection in mouse models of neuropsychiatric disorders.

## Data Availability Statement

The original contributions presented in the study are included in the article/supplementary material, further inquiries can be directed to the corresponding author/s.

## Ethics Statement

The animal study was reviewed and approved by The animal Ethics Committee of the Government of Baden Württemberg, Regierungspräsidium Karlsruhe, (G-101/16).

## Author Contributions

AE: conceptualization, data curation, formal analysis, project administration, supervision, validation, visualization, and writing–original draft, review and editing. BK: methodology, data curation, and formal analysis. CP: conceptualization, project administration, supervision, and validation, review and editing. All authors contributed to the article and approved the submitted version.

## Conflict of Interest

The authors declare that the research was conducted in the absence of any commercial or financial relationships that could be construed as a potential conflict of interest.

## Publisher’s Note

All claims expressed in this article are solely those of the authors and do not necessarily represent those of their affiliated organizations, or those of the publisher, the editors and the reviewers. Any product that may be evaluated in this article, or claim that may be made by its manufacturer, is not guaranteed or endorsed by the publisher.
